# Feasibility and Safety of a Novel Leg Exercise Apparatus for Venous Thromboembolism Prophylaxis after Total Joint Arthroplasty of the Lower Extremities—A Pilot Study

**DOI:** 10.3390/tomography7040061

**Published:** 2021-11-04

**Authors:** Kenta Tanaka, Yukiyo Shimizu, Hiroshi Kamada, Shizu Aikawa, Hajime Mishima, Akihiro Kanamori, Tomofumi Nishino, Masataka Sakane, Naoyuki Ochiai, Masashi Yamazaki

**Affiliations:** 1 Department of Orthopaedic Surgery, Faculty of Medicine, University of Tsukuba, 1-1-1 Tennodai, Tsukuba 305-8575, Ibaraki, Japan; chattymachine2000@yahoo.co.jp (K.T.); hkamada@md.tsukuba.ac.jp (H.K.); hmishima@md.tsukuba.ac.jp (H.M.); kanamori@md.tsukuba.ac.jp (A.K.); nishino@md.tsukuba.ac.jp (T.N.); sakane-m@tsukuba-seikei.jp (M.S.); ochiainaoyuki@ob.md.tsukuba.ac.jp (N.O.); masashiy@md.tsukuba.ac.jp (M.Y.); 2 Department of Orthopaedic Surgery, Nogami Hospital, Tozakimachi 6-8, Tsuchiura 300-0031, Ibaraki, Japan; 3 Department of Rehabilitation Medicine, Faculty of Medicine, University of Tsukuba, 1-1-1 Tennodai, Tsukuba 305-8575, Ibaraki, Japan; 4 Department of Cardiovascular Surgery, Tsukuba Medical Center Hospital, 1-3-1 Amakubo, Tsukuba 305-8558, Ibaraki, Japan; shizu.aikawa@gmail.com; 5 Department of Orthopaedic Surgery, Tsukuba Gakuen Hospital, 2573-1 Kamiyokoba, Tsukuba 305-0854, Ibaraki, Japan; 6 Department of Orthopaedic Surgery, Kikkoman General Hospital, 100 Miyazaki, Noda 278-0005, Chiba, Japan

**Keywords:** leg exercise apparatus, venous thromboembolism prophylaxis, mechanical prophylaxis, arthroplasty, lower extremities

## Abstract

Venous thromboembolism (VTE) is a severe complication in orthopedic surgeries. Herein, we developed a novel leg exercise apparatus (LEX) to encourage postoperative limb movement in bedridden patients to prevent VTE. We aimed to evaluate its feasibility and safety in individuals at risk of VTE. Twenty patients (four men, 16 women) who underwent total joint arthroplasty in the lower extremity were enrolled in this prospective study. Exercise using the LEX was performed for 5 min at 30 cycles/min, four times/day during postoperative days 1–7. Clinical assessments included the evaluation of vital signs, venous ultrasonography, and blood tests within seven days postoperatively, and adverse events (pulmonary embolism and cerebral hemorrhage) were monitored. Overall, 16/20 (80%) patients completed the 7-day exercise regimen. There were no cases of severe adverse events, changes in vital signs, or lower-extremity deep vein thrombosis in patients who performed exercises with the LEX. Thus, the results of this pilot study show that this novel apparatus may be a safe and feasible tool for VTE prophylaxis after joint arthroplasty of the lower extremities.

## 1. Introduction

Venous thromboembolism (VTE) is a medical condition that includes deep vein thrombosis (DVT) and pulmonary embolism (PE) [1,2]. Orthopedic surgery of the lower limbs constitutes a high-risk factor for VTE development: patients undergoing total hip and knee arthroplasty show an incidence of this complication of approximately 0.6–1.5% [3,4]. DVT of the lower limbs usually develops in the calf veins, especially those in the soleus muscle, and is a frequent source of pulmonary emboli [5,6,7].

The guidelines of the American College of Chest Physicians (ACCP) [8] and American Academy of Orthopedic Surgeons (AAOS) [9] recommend mechanical and pharmacological thromboprophylaxis in patients undergoing orthopedic surgery. Specific measures to prevent lower-extremity VTE include mechanical graduated compression stockings (GCS), intermittent pneumatic compression (IPC), and early ambulation, in addition to the administration of antithrombotic agents. Mechanical prophylaxis aims to avoid blood flow stasis, which is considered the main risk factor for DVT in “Virchow’s triad” [10,11]. IPC, which involves applying pressure on the foot and crus, has been proven effective for preventing DVT in many clinical trials [12,13,14]. Additionally, voluntary lower extremity movements, such as those involved in early ambulation and active ankle exercise, are recommended based on their strong benefit on blood flow [5,8,15]. In this regard, using ultrasonography, Sochart and Hardinge [16] evaluated the venous blood flow during active ankle exercises and found a significant increase in the volume and speed during automatic exercises, compared to those on bedrest or when performing externally assisted exercises. Moreover, exercises involving ankle plantar flexion and dorsiflexion combined with varus–valgus angulation have a stronger effect on blood flow than simple ankle plantar flexion or dorsiflexion exercises [16].

However, postoperative patients rarely perform lower extremity exercises voluntarily, even after being encouraged to do so; therefore, GCS and IPC are generally used as perioperative measures for mechanical VTE prophylaxis [12]. Many studies have confirmed the effectiveness of GCS and IPC in preventing thrombosis, but complications, such as lower limb pain or skin disorders, may result from prolonged usage [17].

Therefore, we developed a novel leg exercise apparatus (LEX) to facilitate active leg movement during the early postoperative period [18,19,20,21]. A previous study on healthy adults found that exercising with an LEX was more effective than using IPC alone for improving blood flow [18]. Moreover, other studies conducted by our team have shown that exercising the lower extremities with the LEX was significantly more effective in activating the muscles of the lower limbs and improving blood flow than performing in-bed exercises without an assisting device [19].

The purpose of this study was to evaluate the feasibility and safety of the LEX for VTE prophylaxis after undergoing total joint arthroplasty of the lower limbs.

## 2. Materials and Methods

### 2.1. Participants

This prospective study included 20 patients (four men, 16 women) who underwent total joint arthroplasty of the lower extremities for the first time between November 2014 and January 2016 at the University of Tsukuba Hospital. The operations included 19 total hip arthroplasties (THAs) and one total knee arthroplasty (TKA) (Table 1). The exclusion criteria were as follows: (1) history of DVT in the lower extremity or current anticoagulant treatment; (2) history of cerebral aneurysm or dissecting aortic aneurysm (conditions that may be aggravated by an increase in blood pressure); (3) uncontrolled hypertension; (4) serious conditions (e.g., heart, liver, and/or kidney disease); (5) history of malignancy within five years before the day of consent or suspicion of current malignancy; and (6) confirmed or suspected pregnancy.

### 2.2. Leg Exercise Apparatus

The LEX [18,19,20,21] is a novel device originally developed to perform in-bed lower extremity exercises in patients with impaired mobilization. The device has been designed to enable the performance of exercises in a supine position without diminishing the positive effect of ankle plantar flexion and dorsiflexion on blood flow.

**Figure 1:**
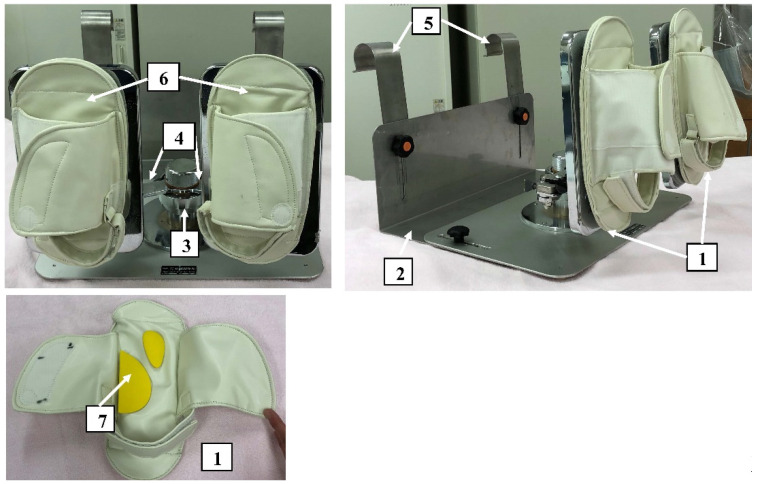
Structure of the leg exercise apparatus. 1: pedals, 2: base, 3: main shaft, 4: arms, 5: hooks, 6: sole, 7: arch supports placed inside the sole.

It is equipped with two (left/right) pedals and a movement control mechanism. The movement control mechanism comprises the base, main shaft, arm, pedal adjuster, and hooks, which are made of stainless-steel (Figure 1). The base is placed on the bed, on which the patient lies, and it is fixed to the backboard of the bed using hooks. The main shaft is placed at the center of the base. A straight arm is placed orthogonally at the top end of the main shaft, and the pedals are attached to both ends of this arm. Since the arm rotates with the main shaft as the center, if the pedal on one side is pressed, the pedal on the other side moves. The foot is fixed to the pedal using a leather sole. The pedal allows ankle movements of up to 30° dorsiflexion, 60° plantar flexion, 30° subtalar inversion, and 20° eversion as the arm rotates. No extra load is added. The LEX does not inhibit ankle movement, as in healthy individuals; the normal ankle movement ranges are 20° dorsiflexion, 45° plantar flexion, 30° varus, and 20° valgus. Moreover, as there is no frictional resistance between the leg and the bed, the device enables a smooth exercise performance that engages the knee and hip joints. Arch supports are placed inside each sole, enabling pressure to be applied to the plantar venous plexus as the pedals are pressed (Figure 1). We intended to maintain a knee flexion position to allow contraction of the soleus, the most frequent site of DVT incidence in the lower leg [5,6], to be predominant over that of the gastrocnemius; usually, with the knee in the extension position, the gastrocnemius contracts while the ankle plantar flexor bends more than the soleus. Exercising with the LEX is presented in detail in Video S1.

Finally, based on the findings of Sochart and Hardinge [16], the design of the LEX included pedals that enabled complex exercises involving subtalar eversion or inversion in addition to dorsiflexion and plantar flexion.

### 2.3. Primary Outcomes

The primary aims of the study were to determine the feasibility and safety of performing exercises using the LEX. For this purpose, we measured the degree of pain and fatigue after the first LEX exercise and the effect on circulatory dynamics related to the use of the LEX. Pain and fatigue were assessed using the visual analog scale (VAS) and the Borg scale [22], respectively (Table 2). The pain was measured using a specialized scale, and fatigue was assessed using a questionnaire after the first LEX exercise. The pulse rate and blood pressure were assessed before and after the first LEX exercise on the operation day, and the blood oxygen saturation level (SpO_2_) was monitored during exercise.

Safety was assessed by registering the adverse events related to the LEX. Adverse events were defined as any medically unfavorable event observed during the study period. Pulmonary embolism, cerebral hemorrhage, and other fatal complications were considered severe adverse events. The safety standard in this study was set as the non-occurrence of such severe adverse events.

### 2.4. Secondary Outcome

The secondary outcome was the occurrence of DVT assessed using ultrasonography of the lower extremities and blood tests, including the D-dimer levels, both performed within seven days postoperatively.

### 2.5. Intervention

**Figure 2:**
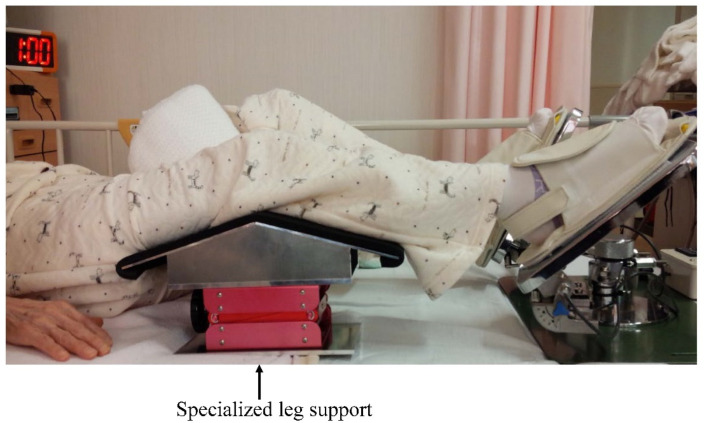
Exercise position using the leg exercise apparatus with a specialized leg support.

Exercise was performed using the LEX device placed as explained above; the body position was adjusted using a specialized leg support (Figure 2) to attain a knee flexion angle of 30° in the supine position.

The pace was set at 30 times/min. Exercise sessions lasted for 5 min and were repeated four times/day; in this regard, the study by Yamashita et al. [23], which investigated assisted exercise involving dorsiflexion and plantar flexion of the foot in postoperative patients, was used as a reference. Exercises were performed during postoperative days 1–7. The first LEX exercise was performed for every participant at 2 h after returning from the operating room (Video S1).

All patients participated in preoperative practice exercises using the LEX until they were comfortable with the device in order to ensure appropriate postoperative use. A physician supervised the first postoperative exercise session and provided instructions to conduct the exercise correctly to avoid inappropriate movements or contraindicated positions.

Compliance with the exercise was confirmed through a counter included in the device, which recorded the number of times the pedals were pressed.

### 2.6. Perioperative Management and Assessment

Assessments and exercises were conducted according to the procedure shown in Table 3. Edoxaban was used in one patient with a high body mass index and in another who underwent TKA, according to the risk analysis of VTE. Thromboprophylactic measures, such as mechanical and pharmacological methods, are presented in Table 1.

IPC continuation was determined according to the performance of activities of daily living (ADLs), as assessed using the Barthel index (BI) [24] (Table 4). In patients who could move in a wheelchair almost independently (BI: 5; movement ability: slight assistance required), IPC was used only at night to avoid limiting the ADLs. IPC use was stopped when the BI value reached 10 points (ability to walk) and when the patient could walk approximately 45 m using a walking assistance device. IPC was detached during exercise with the LEX. GCS was worn during the entire 7-day period. The risk of thromboembolism was assessed based on the Japanese Circulation Society Joint Working Group 2009 guidelines [5].

### 2.7. Cancellation Criteria

If any of the following events occurred during the study period, the patient was excluded, and the specific reason and other relevant data were recorded in the case report: (1) adverse events that rendered the continuation of the study difficult; (2) request from the patient to opt-out of the study; (3) withdrawal of consent for participation (in this case, all data would be excluded); (4) confirmed critical or continued noncompliance to the study plan; and (5) other reasons that the physician deemed sufficient for cancellation. The physician recorded all the content of the survey that was conducted until cancellation.

This study was approved by the Institutional Review Board of the University of Tsukuba Hospital, Japan (approval No.: H17-18; date of registration: 3 June 2013). Based on Ethics Committee recommendations, all patients provided consent for participation and publication, both verbally and in written form.

## 3. Results

Overall, 16 out of 20 patients completed the 7-day exercise regimen, while four patients dropped out. All four patients who dropped out had undergone THA; three of them refused to initiate exercise because of leg pain related to surgery, and the remaining one stopped exercising because of physical difficulties after two days. No severe adverse events occurred during the study period.

In the first exercise session (on the day of operation), the average pain and fatigue scores were 38.5 mm (range, 0–85 mm) and 13 (range, 7–19), respectively.

**Figure 3:**
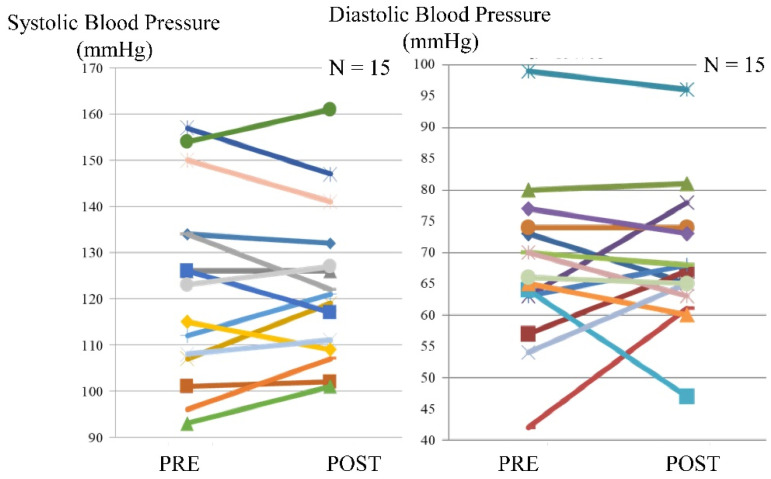
Blood pressure before and after exercise using the leg exercise apparatus.

Blood pressure was assessed in 15 patients. An increase in systolic blood pressure before or after exercise was observed in seven patients, while an increase in diastolic blood pressure before or after exercise was observed in six patients. The maximum difference between systolic blood pressure before and after exercise was 11 mmHg, whereas, for the diastolic blood pressure, the corresponding value was 19 mmHg (Figure 3).

**Figure 4:**
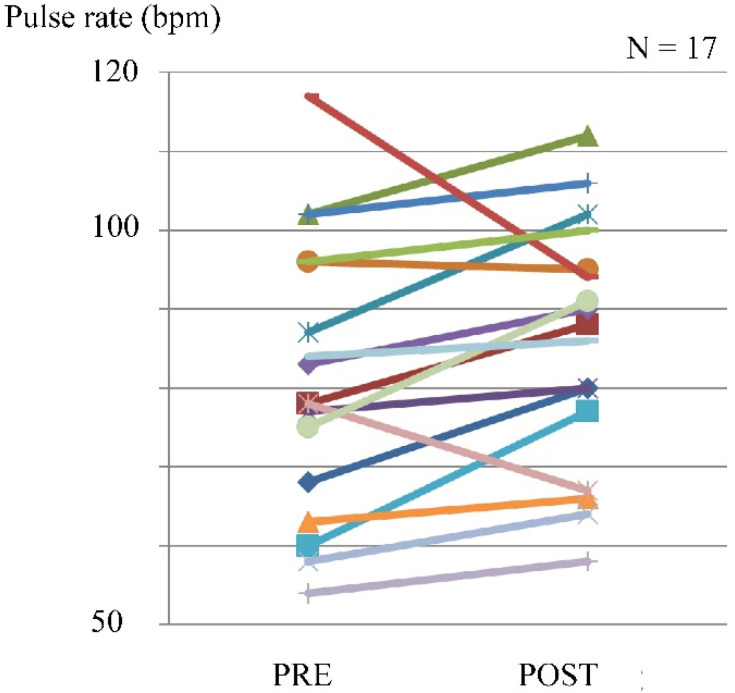
Pulse rate before and after exercise using the leg exercise apparatus.

The pulse rate was evaluated in 17 cases. The pulse rate increased in 14 patients, with a maximum of 117 beats/min (bpm), and the maximum difference before and after exercise was 17 bpm (Figure 4).

**Figure 5:**
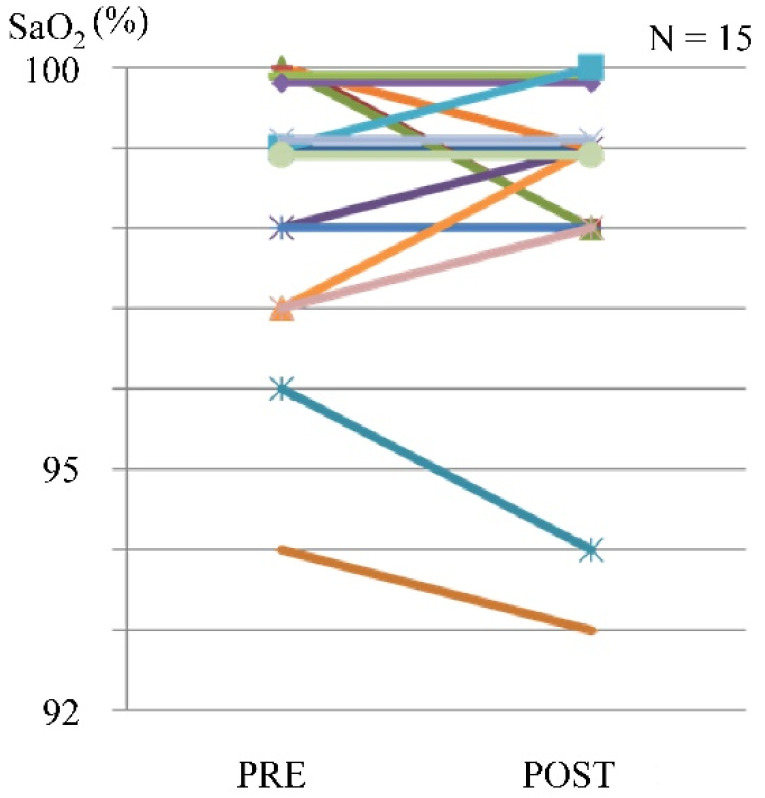
Blood oxygen saturation level before and after exercise using the leg exercise apparatus.

In some cases, a maximum reduction of 2% in SpO_2_ was observed, but there were no complaints of dyspnea (Figure 5).

**Figure 6:**
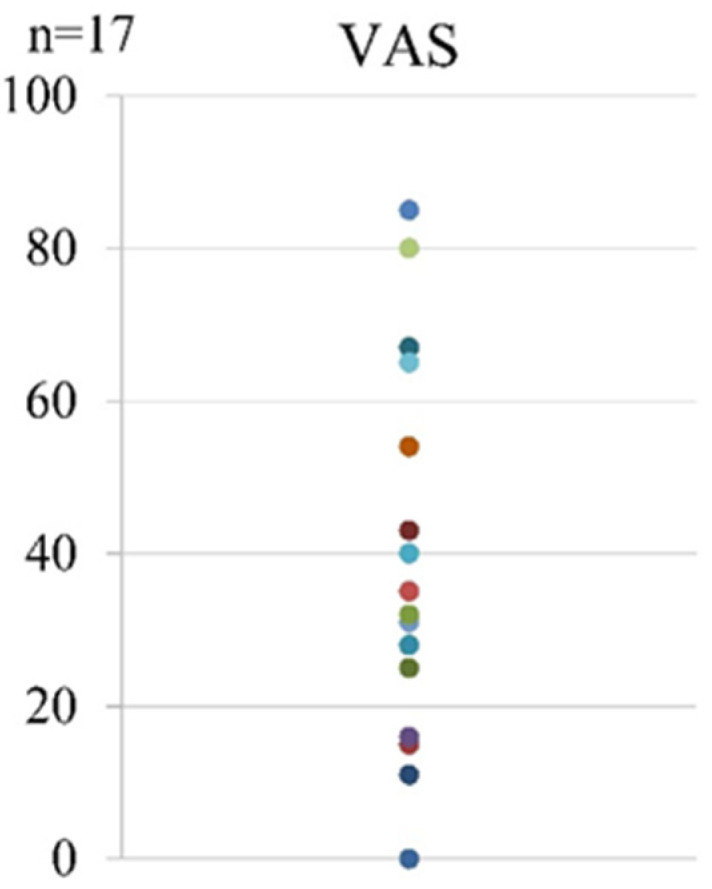
The VAS scores after the first LEX exercise. LEX, leg exercise apparatus; VAS, visual analog scale.

Regarding pain after the first LEX exercise, the mean VAS score was 38.5 (range, 0–85) (Figure 6).

**Figure 7:**
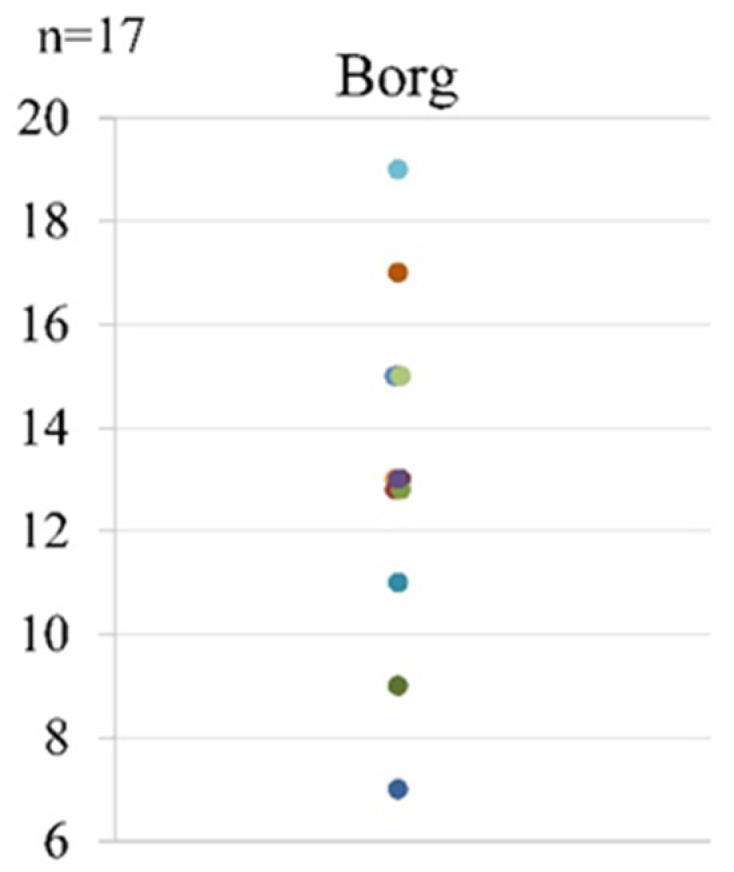
The Borg scale scores after the first LEX exercise. LEX, leg exercise apparatus.

As for fatigue after the first LEX, the mean Borg scale score was 13 (range, 7–19) (Figure 7).

Regarding the secondary outcome, no DVT of the lower extremities was confirmed during the first postoperative week. The average preoperative D-dimer level was 0.85 µg/mL (range, 0.4–2.2), whereas the corresponding value on the first postoperative week was 7.93 µg/mL (range, 2.0–13.8).

## 4. Discussion

In this study, the LEX was successfully used in patients after total joint arthroplasty of the lower extremities, and no severe adverse events occurred. Overall, 80% of patients completed the 1-week protocol. This acceptance rate is comparable to that of IPC, which has been reported to be 81% [17,25].

Regarding the safety of a device that affects circulatory dynamics through exercise, none of the measured values for systolic or diastolic blood pressure, pulse rate, and SpO_2_ exceeded the exercise cancellation standard set by the Japanese Association of Rehabilitation Medicine [26] (Table 5). This finding suggests that it is unlikely that exercising with the LEX could cause any hemodynamic complication. Hence, the LEX exercise may be safely performed even in patients who underwent total joint arthroplasty only 2 h before the LEX procedure. Thus, LEX might be an optimal method for postoperative patients.

According to the ACCP [8] and AAOS [9] guidelines, the main aim of thromboprophylaxis is the prevention of lethal or symptomatic VTE; as opposed to previous guidelines, indications for anticoagulant therapy are more restricted because of the risk of major bleeding complications and cost-effectiveness issues. Specifically, the AAOS guidelines clarify that pharmacological prophylaxis is only recommended for patients who are not susceptible to bleeding after surgery [27]. This guideline recommends mechanical prevention for asymptomatic VTE in patients with a high risk of bleeding.

Sashi et al. [28] examined the VTE rates in total hip and knee arthroplasty between 2002 and 2011 and found that, despite a slight decrease in VTE incidence related to both surgeries, the PE rates remained stable. In detail, the overall median DVT and PE incidences in the cited study were 0.40 and 0.23 in primary THA and 0.62 and 0.34 in primary TKA, respectively. Chan et al. [29] conducted a systematic review of randomized controlled trials comparing the rates of VTE and bleeding due to pharmacological prophylaxis after THA or TKA; they found an overall VTE rate of 0.99%, which was similar to that reported in previous studies. However, the postoperative bleeding rate was 3.44%, which is more than three times the VTE rate. Fuji et al. [4] examined the development, prophylaxis, and treatment of VTE and bleeding events in 36,947 patients who had undergone orthopedic surgeries of the lower extremities from 2008 to 2013 using a healthcare database. They reported that the incidences of DVT, PE, and bleeding were 1.3%, 0.2%, and 1.0% for TKA and 0.9%, 0.2%, and 1.1% for THA, respectively. Therefore, the rate of bleeding complications due to antithrombotic therapy was higher than that of PE in both studies.

Tsuda et al. [30] reported the incidence of DVT solely using mechanical prophylaxis. Their study showed that, among 184 cases of hip surgery receiving only IPC and GCS (patients with trauma were excluded), 5% of them were diagnosed with distal thromboses according to the ultrasonic tests conducted on postoperative week 3.

Considering the relatively high incidence of bleeding events, the current opinion is that individual risk analyses for VTE are needed [3,31]. Thus, we consider designing study protocols using the LEX as mechanical prophylaxis according to VTE risk stratification, in addition to pharmacological prophylaxis. 

According to recent studies focusing on fast-track THA and TKA, there is a low risk of VTE after surgery [32,33]. It appears that early ambulation after the surgery is crucial, and prolonged pharmacological prophylaxis is not required. In minimally invasive surgeries, 7-day prophylaxis using the LEX may be unnecessary. However, many patients experienced difficulty getting out of bed in the early stage after the surgery. The LEX device may motivate patients to move their legs actively and consequently encourage early ambulation postoperatively.

Finally, given the current pandemic, it is worth noting that coronavirus disease 2019 significantly increases the risk of VTE development [34,35]. Moreover, self-isolation periods prior to lower limb arthroplasty may increase the thrombotic risk further [31]. In a period in which close contact should be reduced to a minimum, mechanical devices that enable self-managed exercise, such as the LEX, may be valuable for safe DVT prophylaxis and appropriate rehabilitation. During the pandemic, this assistive device for patient rehabilitation would be useful. LEX may be able to prevent close contact between medical staff and patients during rehabilitation. Furthermore, the LEX might take on new significance during this pandemic. 

There were some limitations to this study. First, the sample size was too small to evaluate effectiveness in the prevention of VTE. However, since DVT after arthroplasty of the lower limbs is very common, it may be a significant finding that no case of DVT was observed in our cohort. Second, we analyzed the use of the LEX in patients who were independent in their ADLs before surgery and, therefore, could resume ambulation relatively soon after surgery. Third, patients in our study received other prophylactic methods, such as IPC and anticoagulants, which could account for the absence of VTE cases. We propose to evaluate the efficacy of the LEX in combination with established strategies to prevent VTE development in bedridden patients.

This is a pilot study that aimed to determine the optimal protocol for VTE prophylaxis using the LEX. We are considering further large-scale studies on the clinical application of the LEX to various populations of bedridden patients, such as those with spinal disease and cancer.

## 5. Conclusions

In this study, the use of the LEX after total joint arthroplasty of the lower extremities was associated with very good acceptance and no severe adverse events. Additionally, no cases of DVT occurred postoperatively. Our pilot results suggest that a study protocol of lower extremity exercises using the LEX for VTE prophylaxis was safe and feasible in this group of patients. We believe that the LEX may be useful in patients with high VTE and bleeding risks, such as those with malignancies or those undergoing spine surgery, wherein pharmacological prophylaxis may be of concern. Additionally, the LEX has potential educational value for patients and medical experts as it could be used to teach the importance of active leg exercises for thromboembolism prophylaxis.

## Figures and Tables

**Table 1:** Background of the subjects.

Subject	Sex	Age (y)	Height (cm)	Weight (kg)	BMI (kg/m^2^)	Surgery	GCS	IPCD	Pharmacological Therapy
1	Male	48	161.0	77.4	29.9	THA	+	* −	Edoxaban
2	Female	70	158.0	54.8	22.0	THA	+	+	-
3	Female	56	153.0	45.6	19.5	THA	+	+	-
4	Female	63	151.1	62.0	27.2	TKA	+	+	Edoxaban
5	Female	43	156.0	66.0	27.1	THA	+	+	-
6	Female	38	155.7	53.4	22.0	THA	+	+	-
7	Female	61	151.8	56.2	24.4	THA	+	+	-
8	Male	38	168.5	76.8	27.0	THA	+	+	-
9	Female	69	145.0	52.8	25.1	THA	+	+	-
10	Female	75	141.0	39.0	19.6	THA	+	+	-
11	Female	59	148.1	50.2	22.9	THA	+	+	-
12	Female	72	131.5	36.6	21.2	THA	+	+	-
13	Female	68	155.7	53.0	21.9	THA	+	+	-
14	Female	55	155.0	49.4	20.6	THA	+	+	-
15	Female	80	146.3	45.0	21.0	THA	+	+	-
16	Female	71	151.0	63.0	27.6	THA	+	+	-
17	Female	69	148.6	62.8	28.4	THA	+	+	-
18	Male	56	168.9	82.8	29.0	THA	+	+	-
19	Female	53	158.4	50.8	20.2	THA	+	+	-
20	Male	67	151.4	70.7	30.8	THA	+	+	-

BMI, body mass index; GCS, graduated compression stockings; IPCD, intermittent pneumatic compression device; THA, total hip arthroplasty. * Refusal by patient.

**Table 2:** The Borg scale: a subjective evaluation of the degree of fatigue.

Borg Scale	
6	
7	Very, very light
8	
9	Very light
10	
11	Fairly light
12	
13	Somewhat hard
14	
15	Hard
16	
17	Very hard
18	
19	Very, very hard
20	

**Table 3:** Procedure characteristics.

Preoperation	Informed Consent D-Dimer Ultrasonography Practice Exercise Using the LEX
Operation day	THA/TKA
	Exercise using the LEX 2 h after surgery and at 5 p.m. with monitoring of ECG, SpO2, and blood pressure VAS·Borg scale
Postoperative day 1–7	Exercise using the LEX at 7 a.m., 10 a.m., 1 p.m., and 5 p.m. VAS·Borg scale
Day 7	D-dimer Ultrasonography

LEX, leg exercise apparatus; SpO_2_, blood oxygen saturation level; VAS, visual analog scale; ECG, electrocardiography; THA, total hip arthroplasty; TKA, total knee arthroplasty.

**Table 4:** The Barthel index for activities of daily living.

FEEDING		
0 = unable		
5 = needs help with cutting, spreading butter, etc., or requires a modified diet		
10 = independent		
BATHING		
0 = dependent		
5 = independent (or in shower)		
GROOMING		
0 = needs help with personal care		
5 = independent face/hair/teeth/shaving (implements provided)		
DRESSING		
0 = dependent		
5 = needs help but can do about half unaided		
10 = independent (including buttons, zips, laces, etc.)		
BOWELS		
0 = incontinent (or needs to be given enemas)		
5 = occasional accident		
10 = continent		
BLADDER		
0 = incontinent or catheterized and unable to manage alone		
5 = occasional accident		
10 = continent		
TOILET USE		
0 = dependent		
5 = needs some help but can do something alone		
10 = independent (on and off, dressing, wiping)		
TRANSFERS (BED TO CHAIR AND BACK)		
0 = unable, no sitting balance		
5 = major help (one or two people, physical), can sit		
10 = minor help (verbal or physical)		
15 = independent		
MOBILITY (ON LEVEL SURFACES)		
0 = immobile or <50 yards		
5 = wheelchair independent, including corners, >50 yards		
10 = walks with the help of one person (verbal or physical) >50 yards		
15 = independent (but may use any aid; for example, stick) >50 yards		
STAIRS		
0 = unable		
5 = needs help (verbal, physical, carrying aid)		
10 = independent		

**Table 5:** Stop exercise criteria in this study.

	Stop Exercise Criteria	Present Study
Systolic blood pressure	Increasing >40 mmHg	Increasing 12 mmHg
Diastolic blood pressure	Increasing >20 mmHg	Increasing 19 mmHg
Pulse rate	Over 140 bpm	Maximum of 112 bpm
SpO_2_	Moderate breathing difficulties	No breathing difficulties
The criteria were set according to the guidelines for safety management and promotion in rehabilitation medicine, proposed by the Japanese Association of Rehabilitation Medicine [26]. SpO_2_, blood oxygen saturation level; bpm, beats/min		

## Data Availability

Data are contained within the article or supplementary material.

## Supplementary Materials

The following are available online at https://www.mdpi.com/article/10.3390/tomography7040061/s1, Video S1. This video shows a participant performing the first exercise using the leg exercise apparatus (LEX) after total hip arthroplasty. The LEX enables users to perform combined leg motion, including hip and knee flexion and extension and ankle dorsi-plantar flexion, inversion, and eversion.
